# Prediction of future cardiovascular disease with an equation to estimate apolipoprotein B in patients with high cardiovascular risk: an analysis from the TNT and IDEAL study

**DOI:** 10.1186/s12944-017-0549-8

**Published:** 2017-08-22

**Authors:** You-Cheol Hwang, Hong-Yup Ahn, Ki Hoon Han, Sung-Woo Park, Cheol-Young Park

**Affiliations:** 1 Division of Endocrinology and Metabolism, Department of Medicine, Kyung Hee University School of Medicine, Kyung Hee University Hospital at Gangdong, Seoul, South Korea; 2 Department of Statistics, Dongguk University-Seoul, Seoul, South Korea; 3 Department of Cardiology, University of Ulsan College of Medicine, Asan Medical Center, Seoul, South Korea; 4 Division of Endocrinology and Metabolism, Department of Internal Medicine, Sungkyunkwan University School of Medicine, Kangbuk Samsung Hospital, 29 Saemunan-ro, Jongno-gu, 03181 Seoul, Republic of Korea

**Keywords:** LDL cholesterol, Non-HDL cholesterol, Apolipoprotein B, Apolipoprotein B equation, Major cardiovascular event

## Abstract

**Background:**

Apolipoprotein B (apoB) is known to be a more powerful predictor of cardiovascular disease than conventional lipids. We aimed to determine the clinical relevance of a newly developed equation to estimate serum apoB levels based on total cholesterol, HDL cholesterol, and triglycerides in patients with high cardiovascular risk.

**Methods:**

The occurrence of a major cardiovascular event (MCVE) was assessed using the data from the Treating to New Targets (TNT) and Incremental Decrease in End points through Aggressive Lipid lowering (IDEAL) trials.

**Results:**

Pooled analysis of these two data sets showed that both directly-measured apoB (HR per 1-SD (95% CI): 1.16 (1.11–1.21), *P* < 0.001) and apoB estimated from the eq. (HR per 1-SD (95% CI): 1.14 (1.09–1.19), *P* < 0.001) were significantly associated with the development of a future MCVE. Prediction of MCVEs by the apoB eq. (C statistic 0.650) was nearly identical to that of directly-measured apoB (0.651). In addition, the net reclassification indices indicated no difference in the prediction of MCVEs between models including the apoB equation and directly-measured apoB (1% (−1.3–4.0), *P* = 0.31).

**Conclusions:**

Our equation to predict apoB levels showed MCVE risk prediction comparable to directly-measured apoB in high risk patients with previous coronary heart disease.

## Background

Recent meta-analysis based on large-scale epidemiologic studies has shown that the measured apolipoprotein B (apoB) level is a more powerful predictor of ischemic cardiovascular events than calculated low-density lipoprotein (LDL) cholesterol level [1]. Moreover, on-treatment levels of non-high density lipoprotein (HDL) cholesterol and apoB levels have been shown to be more strongly associated with major cardiovascular events (MCVEs) than calculated LDL cholesterol levels in patients receiving statin therapy [2].

Despite the potential advantage of apoB over LDL cholesterol for the assessment of cardiovascular risk, measurement of apoB levels are not generally recommended. We recently developed a novel equation to calculate apoB levels from serum lipid parameters including total cholesterol, triglyceride, and HDL cholesterol levels. These parameters are also used for estimating LDL cholesterol levels, i.e. Friedewald’s equation [3]. We previously demonstrated that apoB levels calculated by our novel equation were similar to directly-measured apoB levels, not only for the whole Korean study population, but also for clinically relevant subgroups, including patients with diabetes, atherogenic dyslipidemia, and those being treated with lipid-lowering agents, regardless of their serum triglyceride levels [4]. In addition, apoB determined by our equation was independently associated with future development of cardiovascular outcomes, and the predictive ability for incident cardiovascular disease (CVD) was superior to LDL cholesterol and comparable to non-HDL cholesterol in a prospective, community-based Korean cohort [5].

However, it remains uncertain whether our novel apoB equation can be applied to other ethnicities or whether it has clinical relevance as a predictor for the development of CVD in patients with high risk for future CVD. Therefore, the aim of this study was to validate our novel apoB equation and to compare the clinical relevance of LDL cholesterol, non-HDL cholesterol, and directly-measured apoB levels using data from prospective and randomized clinical trials of the Treating to New Targets (TNT) and Incremental Decrease in End points through Aggressive Lipid lowering (IDEAL) studies [6, 7].

## Methods

### Study population

A detailed description of the TNT and IDEAL studies has been published previously [6, 7]. Both TNT and IDEAL are prospective and randomized multicenter secondary prevention trials proving the increased efficacy of high-dose statin treatment compared to usual-dose statin treatment. A total of 10,001 patients in the TNT trial with stable coronary heart disease were found to have LDL cholesterol levels <3.4 mmol/L after 8 weeks of atorvastatin treatment (10 mg/day) prior to the randomization (‘baseline’). They were subsequently assigned to receive either 10 mg or 80 mg atorvastatin per day. During the follow-up period (median 4.9 years), mean LDL cholesterol levels were maintained at 2.6 mmol/L and 2.0 mmol/L in the 10 mg and 80 mg groups, respectively. In IDEAL trial, 8888 patients with previous myocardial infarction were randomly assigned to receive either high-dose atorvastatin (80 mg/day) or usual-dose simvastatin (20 mg/day) and were followed up for a median of 4.8 years. Mean LDL cholesterol levels during treatment were 2.7 mmol/L in the simvastatin group and 2.1 mmol/L in the atorvastatin group. In the present study, we analyzed both separated and combined data of the TNT and IDEAL trials. In the current study, a total of 9785 subjects in TNT and 8880 subjects in IDEAL were analyzed after excluding subjects without available apoB or lipids levels to calculate estimated apoB.

### Laboratory measurements

Total cholesterol, HDL cholesterol, and triglyceride levels were determined by standard methodologies. LDL cholesterol was calculated using Friedewald’s formula [3] or measured directly when the triglyceride level was 4.5 mmol/L or higher. Non-HDL cholesterol was calculated by subtracting HDL cholesterol from total cholesterol. The serum concentration of apoB was determined by immunonephelometry (Behring Nephelometer BNII, Marburg, Germany).

In the case of TNT data, we analyzed the baseline and 1-year on-treatment lipid and apoB levels. In the case of IDEAL, the same lipid parameters were analyzed at baseline, month 3, and years 1, 2, 3, 4, and 5. In cases of pooled data, we analyzed lipid parameters at baseline and month 12.

### Development of an equation to estimate apoB levels

A detailed development method of the apoB equation has been published previously [4]. We developed two apoB equation models. One is a simple model (model 1) that is valid only for a limited range of triglycerides. The structure of model 2 is essentially identical to that of model 1, but it applies regression coefficients adjusted by the cutoff value of triglycerides. The equation from model 2 can be applied regardless of triglyceride ranges, and it was shown that the triglyceride cutoff value of 3.05 mmol/L had the lowest Bayesian information criterion score. Therefore, essentially no difference in performance was noted between the two models when the serum triglyceride level was <3.05 mmol/L, and model 2 provided a closer estimate of directly-measured apoB levels than model 1 when triglyceride levels were higher than 3.05 mmol/L. In this study, we used model 2 for apoB calculation.

### Outcome definition

In the present analysis, the occurrence of MCVEs was selected as an outcome measurement. MCVE was defined as either death from coronary heart disease, nonfatal myocardial infarction, resuscitation after cardiac arrest, or fatal or nonfatal stroke.

### Statistical methods

Data were expressed as the mean ± SD or SE. Survival analyses were used to validate serum LDL cholesterol, non-HDL cholesterol, and apoB levels and the calculated apoB values as potential predictors of future MCVEs. In order to compare these measurements, we calculated hazard ratios (HR) for each 1-SD increment using the multivariate Cox proportional hazards model. Harrell’s C statistic and net reclassification improvement statistic were used to compare the performance of the models in predicting future MCVEs. Analyses were performed using R version 2.14.2 (http://www.r-project.org). *P*-values of <0.05 were considered statistically significant.

## Results

Table 1 shows baseline characteristics of lipid parameters. In the 9785 TNT subjects, after 8 weeks of treatment with 10 mg atorvastatin per day, directly measured mean apoB levels were 1.11 g/L and mean estimated apoB levels were 0.87 g/L. In the 8880 IDEAL subjects, mean directly measured and estimated apoB levels were 1.19 g/L and 1.01 g/L or 1.02 g/L, respectively. Across all four randomized groups, serum LDL cholesterol and non-HDL cholesterol levels ranged from 2.52 mmol/L to 3.15 mmol/L and 3.30 mmol/L to 3.90 mmol/L in the TNT and IDEAL trials.

**Table 1:** Baseline characteristics

Variable	TNT		IDEAL	
	ATV 10 mg	ATV 80 mg	SIMV 20-80 mg	ATV 80 mg
N	4907	4878	4445	4435
Total cholesterol (mmol/L)	4.53 (0.67)	4.52 (0.65)	5.07 (1.01)	5.09 (1.02)
Triglyceride (mmol/L)	1.71 (0.90)	1.71 (0.86)	1.66 (0.86)	1.71 (0.93)
HDL cholesterol (mmol/L)	1.21 (0.29)	1.22 (0.29)	1.19 (0.32)	1.19 (0.31)
LDL cholesterol (mmol/L)	2.54 (0.50)	2.52 (0.49)	3.14 (0.90)	3.15 (0.90)
Non-HDL cholesterol (mmol/L)	3.32 (0.64)	3.30 (0.63)	3.87 (1.00)	3.90 (1.01)
Apolipoprotein B (g/L)	1.11 (0.20)	1.11 (0.19)	1.19 (0.32)	1.19 (0.32)
Estimated apoB (g/L)	0.87 (0.16)	0.87 (0.16)	1.01 (0.25)	1.02 (0.25)

Data are expressed as mean (SD), derived from baseline values in TNT or IDEAL

*ATV* atorvastatin, *SIMV* simvastatin

The analysis of TNT data showed that apoB levels calculated by our equation were found to be significantly lower than the measured apoB levels, and the percentage of difference between calculated and measured apoB levels was 21.6% at baseline and 20.4% and 23.1% in the 10 mg/day and 80 mg/day atorvastatin groups, respectively after 1 year of treatment. When data from both 10 mg/day and 80 mg/day atorvastatin groups were combined, age- and sex-adjusted Cox proportional hazard regression analyses showed that non-HDL cholesterol (HR per 1-SD (95% CI): 1.21 (1.13–1.29), *P* < 0.001) and directly-measured apoB (HR per 1-SD (95% CI): 1.20 (1.12–1.28), *P* < 0.001) levels were clearly associated with the risk of developing MCVEs within 1-year of initiating treatment with atorvastatin. The predictive power of LDL cholesterol levels seemed to be less potent than that of other measured lipid parameters (HR per 1-SD (95% CI): 1.15 (1.08–1.22), *P* < 0.001). Interestingly, the calculated apoB values could predict future MCVE as effectively as measured apoB levels (HR per 1-SD (95% CI): 1.21 (1.13–1.29), *P* < 0.001) (Table 2).

**Table 2:** Lipid parameters and major cardiovascular events in TNT

Variable		Baseline	1 year
N	ATV 10 mg	4907	4707
	ATV 80 mg	4878	4675
LDL cholesterol (mmol/L)	ATV 10 mg	2.54 (0.01)	2.61 (0.01)
	ATV 80 mg	2.52 (0.01)	1.94 (0.01)
	HR (95% CI)	1.07 (1.01–1.14)	1.15 (1.08–1.22)
Non-HDL cholesterol (mmol/L)	ATV 10 mg	3.32 (0.01)	3.41 (0.01)
	ATV 80 mg	3.30 (0.01)	2.61 (0.01)
	HR (95% CI)	1.15 (1.08–1.22)	1.21 (1.13–1.29)
Apolipoprotein B (g/L)	ATV 10 mg	1.11 (0.0)	1.13 (0.0)
	ATV 80 mg	1.11 (0.0)	0.91 (0.0)
	HR (95% CI)	1.13 (1.06–1.21)	1.20 (1.12–1.28)
Estimated apoB (g/L)	ATV 10 mg	0.87 (0.0)	0.90 (0.0)
	ATV 80 mg	0.87 (0.0)	0.70 (0.0)
	HR (95% CI)	1.13 (1.06–1.21)	1.21 (1.13–1.29)

Data are expressed as mean (SE) or HR per 1-SD (95% CI)

Adjusted for age and sex

*ATV* atorvastatin

The analysis of IDEAL data showed a similar trend in that calculated apoB values were found to be 15.1% and 14.3% lower than measured apoB values. During the statin treatment periods from 3 months to 5 years, the mean difference between directly measured and calculated apoB levels was 16.9% in the simvastatin group and 16.3% in the atorvastatin group. In age- and sex-adjusted Cox proportional hazard regression analyses, the risk of future MCVE ranged from 1.13 to 1.18 for directly measured apoB levels and from 1.11 to 1.17 for calculated apoB values throughout the treatment period up to four years. Moreover, LDL cholesterol levels seemed to less powerfully predict future MCVE (HR between 1.08–1.13) as observed in TNT data (Table 3).

**Table 3:** Lipid parameters and major cardiovascular events in IDEAL

Variables		Baseline	12 week	1 year	2 year	3 year	4 year	5 year
N	SIMV	4445	4371	4290	4168	4033	3930	775
	ATV	4435	4334	4198	4099	3985	3861	758
LDL cholesterol (mmol/L)	SIMV	3.14 (0.01)	2.71 (0.01)	2.64 (0.01)	2.68 (0.01)	2.75 (0.01)	2.68 (0.01)	2.58 (0.02)
	ATV	3.15 (0.01)	2.01 (0.01)	2.05 (0.01)	2.12 (0.01)	2.22 (0.01)	2.16 (0.01)	2.07 (0.03)
	HR	1.14 (1.07–1.20)	1.09 (1.03–1.16)	1.12 (1.05–1.19)	1.13 (1.06–1.20)	1.10 (1.03–1.18)	1.08 (1.01–1.16)	1.06 (0.91–1.25)
Non-HDL cholesterol (mmol/L)	SIMV	3.87 (0.02)	3.41 (0.01)	3.33 (0.01)	3.36 (0.01)	3.43 (0.01)	3.37 (0.01)	3.26 (0.03)
	ATV	3.90 (0.02)	2.59 (0.01)	2.63 (0.01)	2.70 (0.01)	2.79 (0.01)	2.74 (0.01)	2.67 (0.03)
	HR	1.15 (1.08–1.21)	1.10 (1.04–1.17)	1.15 (1.08–1.22)	1.18 (1.11–1.25)	1.12 (1.04–1.20)	1.11 (1.03–1.19)	1.07 (0.91–1.25)
Apolipoprotein B (g/L)	SIMV	1.19 (0.0)	1.05 (0.0)	1.07 (0.0)	1.03 (0.0)	1.06 (0.0)	1.09 (0.0)	1.08 (0.01)
	ATV	1.19 (0.0)	0.80 (0.0)	0.84 (0.0)	0.82 (0.0)	0.86 (0.0)	0.90 (0.0)	0.91 (0.01)
	HR	1.15 (1.09–1.22)	1.13 (1.06–1.19)	1.17 (1.10–1.24)	1.18 (1.11–1.26)	1.16 (1.08–1.24)	1.13 (1.05–1.21)	1.07 (0.91–1.26)
Estimated apoB (g/L)	SIMV	1.01 (0.0)	0.90 (0.0)	0.87 (0.0)	0.88 (0.0)	0.90 (0.0)	0.89 (0.0)	0.86 (0.01)
	ATV	1.02 (0.0)	0.69 (0.0)	0.70 (0.0)	0.72 (0.0)	0.74 (0.0)	0.73 (0.0)	0.71 (0.01)
	HR	1.15 (1.09–1.21)	1.11 (1.04–1.17)	1.14 (1.08–1.21)	1.17 (1.10–1.24)	1.12 (1.05–1.20)	1.11 (1.04–1.20)	1.07 (0.91–1.25)

Data are expressed as mean (SE) or HR per 1-SD (95% CI)

Adjusted for age and sex

*ATV* atorvastatin, *SIMV* simvastatin

When baseline and 1-year treatment data of both the TNT and IDEAL trails were combined, predictive powers of non-HDL cholesterol (HR per 1-SD (95% CI): 1.15 (1.10–1.20), *P* < 0.001), directly-measured apoB levels (HR per 1-SD (95% CI): 1.16 (1.11–1.21), *P* < 0.001) and calculated apoB values (HR per 1-SD (95% CI): 1.14 (1.09–1.19), *P* < 0.001) for future MCVE risk were comparable to each other, and LDL cholesterol was found to be less predictive than the aforementioned lipid parameters (HR per 1-SD (95% CI): 1.10 (1.05–1.15), *P* < 0.001) (Table 4).

**Table 4:** Lipid parameters and major cardiovascular events in TNT and IDEAL

Variable	HR (95% CI)	*P*
LDL cholesterol	1.10 (1.05–1.15)	<0.001
Non-HDL cholesterol	1.15 (1.10–1.20)	<0.001
Apolipoprotein B	1.16 (1.11–1.21)	<0.001
Estimated apoB	1.14 (1.09–1.19)	<0.001

Data are expressed as HR per 1-SD (95% CI)

Adjusted for the effects of study, age and sex

Table 5 presents the C statistics and net reclassification improvement statistics comparing the models of LDL cholesterol, non-HDL cholesterol, directly-measured apoB, and estimated apoB. The overall performance of the models with the aforementioned four lipid parameters was similar by C statistics: LDL cholesterol (0.648), non-HDL cholesterol (0.650), estimated apoB (0.650), and directly-measured apoB (0.651). We next compared each model of LDL cholesterol, non-HDL cholesterol, directly-measured apoB, and estimated apoB. As a result, directly-measured apoB significantly outperformed LDL cholesterol for prediction of a future MCVE (3.5% (0.3–6.4), *P* = 0.007); however, no difference was observed between directly-measured apoB and estimated apoB in predicting future MCVEs. In addition, although it did not reach statistical significance, estimated apoB showed a trend towards better prediction of a future MCVE compared with LDL cholesterol (3.2% (−0.3–6.4), *P* = 0.09).

**Table 5:** C statistics and net reclassification improvement statistics of lipid parameters for major cardiovascular events in TNT and IDEAL

Models	C statistics	
LDL cholesterol	0.648 (0.634–0.662)	
Non-HDL cholesterol	0.650 (0.636–0.664)	
Apolipoprotein B	0.651 (0.637–0.665)	
Estimated apoB	0.650 (0.636–0.664)	
Models	Net reclassification improvement statistics	*P*
LDL cholesterol vs. apolipoprotein B	3.5% (0.3–6.4)	0.007
LDL cholesterol vs. estimated apoB	3.2% (−0.3–6.4)	0.09
LDL cholesterol vs. non-HDL cholesterol	3.8% (0.5–6.8)	0.013
Estimated apoB vs. non-HDL cholesterol	5.2% (1.7–10.2)	0.013
Estimated apoB vs. apolipoprotein B	1.0% (−1.3–4.0)	0.31
Non-HDL cholesterol vs. apolipoprotein B	1.1% (−1.4–3.9)	0.29

Adjusted for the effects of study, age, and sex

## Discussion

Currently, LDL cholesterol is regarded as the primary target for the prevention of CVD [8]. However, approximately one half of patients with recurrent acute coronary syndrome had normal cholesterol levels [9], and despite aggressive statin therapy and achieving an LDL cholesterol target goal, a significant number of patients still experience recurrent coronary events [10]. This suboptimal cardiovascular risk prediction using LDL cholesterol prompts the need to identify better lipid markers for assessing cardiovascular risk. In addition, LDL cholesterol levels have been frequently calculated with Friedewald’s formula using total cholesterol, triglyceride, and HDL cholesterol levels; however, this formula cannot be used when triglyceride levels exceed 400 mg/dL [3]. Furthermore, it has been reported that the agreement between calculated and directly measured LDL cholesterol levels decreases as the serum triglyceride concentration increases, even when the triglyceride concentration is less than 400 mg/dL [11, 12].

In this context, the measurement of non-HDL cholesterol or apoB is reasonable because this lipid parameter provides better cardiovascular risk prediction when compared with LDL cholesterol [1, 2, 13, 14]. However, despite the clinical benefits of using apoB over LDL cholesterol for assessing cardiovascular risk, apoB levels are not routinely measured due to additional costs and lag time in obtaining results, which causes delays in appropriate intervention [15]. Therefore, using data set from 78,127 Korean subjects who visited the Health Screening Center, we developed a new equation to estimate apoB levels from total cholesterol, triglyceride, and HDL cholesterol levels that are identical to the lipid parameters used in Friedewald’s formula [3]. Although our study subjects were not a nationally representative population, many of these subjects were employees mandated by Industrial Safety and Health Law in Korea to participate in health examinations, and therefore, our study subjects could represent the characteristics of the general population of South Korea. In this study, the correlation coefficient between the measured and estimated apoB levels was 0.95, and the difference between the measured and estimated apoB level was less than 16 mg/dL for 95% of all subjects. In other words, only 5% of our study subjects exhibited an apoB estimation error greater than 16 mg/dL [4].

In the analysis of TNT data, the percentage of difference between directly-measured and estimated apoB levels was 20.4% after 10 mg atorvastatin treatment and 23.1% after 80 mg atorvastatin treatment. Similarly, in the analysis of IDEAL data, the percentage of difference between these two apoB measurements was 18.7% in the simvastatin group and 16.7% in the atorvastatin group after 1-year treatment. These differences between directly measured and estimated apoB levels were quite large compared to those of our previous study [4]. We could not clearly explain these discrepancies, but some suggestions can be made. First, our equation was developed from lipid parameters of a Korean population; however, the subjects that participated in TNT and IDEAL were almost exclusively Caucasian, and this ethnic difference could account for the difference in directly measured and estimated apoB levels between our previous study and current analysis of TNT and IDEAL. In actuality, it was reported that Asians have lower LDL cholesterol, HDL cholesterol, triglyceride and apoB levels when compared to non-Asians [16]. In addition, South Asians have comparable LDL cholesterol levels to other population, but their LDL particle size tends to be smaller [17]. Furthermore, they not only have lower HDL cholesterol levels, but they also have a higher concentration of small, less-protective HDL particles [18]. However, in our previous study, we validated our equation with a nationally representative US population from NHANES data, and the result showed that measured and estimated apoB levels were 90.5 mg/dL and 93.6 mg/dL, respectively, which indicates that our equation was applicable to ethnicities other than the Korean population [4]. Although these studies included a negligible portion of non-Caucasian subjects (5.9% in TNT and <1% in IDEAL), no differences between directly measured and estimated apoB levels were observed between different ethnic groups in the TNT study (data not shown). Therefore, it appears that relatively large differences between directly measured and estimated apoB levels in the current analysis were not solely due to differences in ethnicity. Second, statins inhibit cholesterol synthesis in the liver and mainly reduce LDL cholesterol in circulation, but the effects of statins on HDL cholesterol and triglycerides are relatively small. Our equation was developed based on a linear regression model that contains total cholesterol, triglycerides, and HDL cholesterol. Different effects of statin on each of these three lipid parameters may affect the accuracy of the equation’s ability to estimate apoB. Only 3.4% of the subjects with equation development data set took lipid-lowering medications [4], but all subjects in the TNT and IDEAL trials took variable doses of statins [6, 7]. Although only 3.4% of subjects were taking lipid-lowering medications, this represents 2650 actual subjects, and the mean estimation error was 2.6 mg/dL (range, −6.3 to 4.4 mg/dL), which may be acceptable in clinical practice [4]. In addition, the changes in estimation error were small after statin treatment in the analysis of IDEAL data. More specifically, the difference between directly measured and estimated apoB levels increased from 15.1% at baseline to a mean of 16.9% in the simvastatin group and from 14.3% at baseline to a mean of 16.3% in the atorvastatin group throughout 5 years of treatment [7]. Therefore, it appears that statin treatment is unlikely to be the cause of the large differences between directly measured and estimated apoB levels in the current analysis. Third, the analytic methods used to measure apoB were different between equation development data and the TNT and IDEAL studies. When we developed an equation, estimated serum apoB concentrations were determined by the immunoturbidometric method, whereas the plasma concentration of apoB was determined by immunonephelometry in the TNT and IDEAL studies. However, it was reported that the bias and imprecision for 22 immunonephelometric and immunoturbidimetric assays ranged from −5.3 to 3.6% and 0.9 to 3.2%, respectively, but accurate results and between-laboratory comparability could be achieved [19]. Another possibility for the difference in estimation error between our previous study and the current TNT and IDEAL analysis may be due to the difference in characteristics of study subjects. Subjects of the equation development group were recruited from the Health Screening Center, and thus, the majority of subjects was relatively healthy and had no previous CVD. In contrast, all TNT and IDEAL patients had previous stable coronary heart disease or myocardial infarction. It was reported that although there is a statistically significant relationship between LDL cholesterol and apoB, the dispersion around the line of identity is remarkable, and plasma apoB was found at various concentrations at a given LDL cholesterol level [20]. Thus, despite LDL cholesterol levels being grossly similar, subjects with insulin resistance, metabolic syndrome, type 2 diabetes, and CVD had higher levels of apoB compared with healthy subjects [21–23]. Similarly, when we compared lipid profiles between subjects from the equation development data and IDEAL study at baseline, total cholesterol levels were almost identical, while estimated LDL cholesterol levels were 114 mg/dL in subjects in the equation development group and 121 mg/dL in IDEAL subjects at baseline. However, directly measured apoB levels were strikingly different in these two data sets. Directly measured apoB levels were found to be 96 mg/dL in equation development subjects and 119 mg/dL in IDEAL subjects, respectively. Therefore, it appears that the difference between directly measured and estimated apoB levels is mainly due to the difference in lipid profile characteristics. More specifically, TNT and IDEAL subjects had more apoB-containing lipoprotein particles at a given total cholesterol level compared with the healthy general population, which can lead to an estimation error. Therefore, our equation may have some limitation in subjects with CVD who have more apoB-containing lipoprotein particles than those in the general population.

Despite the increased estimation error in the current study, apoB levels estimated by our equation were highly correlated with directly measured apoB (*r* = 0.91 in TNT and *r* = 0.94 in IDEAL, data not shown). This has significant clinical relevance. In this study, estimated apoB in subjects on cholesterol-lowering therapy was associated with future MCVE, and the standardized hazard ratio was similar to those of non-HDL cholesterol and directly measured apoB. In addition, the performance of the equation was valid regardless of duration of statin therapy up to five years in the IDEAL analysis, and the standardized hazard ratio of the apoB equation was identical to that of directly measured apoB after 5 years of statin therapy. Furthermore, Friedewald’s formula can be used only when triglyceride levels are less than 400 mg/dL while our equation can be applied regardless of triglyceride ranges. In addition, we have validated the performance of our equation to predict future CVD in 9001 healthy individuals from the general Korean population [5]. During a mean 8.1 years of follow-up, both non-HDL cholesterol (HR per 1-SD (95% CI); 1.13 (1.05–1.23), *P* = 0.002) and estimated apoB (HR per 1-SD (95% CI); 1.14 (1.05–1.24), *P* = 0.001) showed a similar performance in predicting future CVD independent of age, sex, waist circumference, current smoking, and presence of diabetes and hypertension; however, LDL cholesterol level was not predictive of future CVD (HR per 1-SD (95% CI); 1.07 (0.99–1.16), *P* = 0.08). Furthermore, net reclassification improvement statistics indicated that the apoB equation significantly outperformed LDL cholesterol in predicting future CVD (15.3% (0.08–0.21), *P* < 0.001); however, no difference was noted between estimated apoB and non-HDL cholesterol in predicting future CVD (data not shown).

This study has some limitations. First, although our equation’s ability to predict future MCVE was comparable to that of directly measured apoB there is considerable estimation error between directly measured apoB and estimated apoB. Second, the analytic methods used to measure apoB were different from the TNT and IDEAL studies and our equation development data set. Third, subjects in the TNT and IDEAL studies were uniformly recruited from patients with previous CVD, and all patients received statin therapy. Thus, our equation should be validated in other populations, including type 2 diabetes and other ethnicities.

## Conclusions

In summary, our equation to estimate apoB was predictive of future development of MCVE and had comparable risk prediction to that of directly-measured apoB. Our apoB equation has clinical relevance that outweighs Friedewald’s formula to estimate LDL cholesterol because our equation was at least comparable to Friedewald’s formula in predicting future MCVE. Furthermore, our apoB equation can be applied regardless of the triglyceride range using the same lipid parameters with the Friedewald’s formula.

## Abbreviations

ApoB: Apolipoprotein B
CVD: Cardiovascular disease
HDL: High density lipoprotein
IDEAL: Incremental decrease in end points through aggressive lipid lowering
LDL: Low-density lipoprotein
MCVE: Major cardiovascular event
TNT: Treating to new targets
